# Non-invasive assessment of inflammatory bowel disease activity using a DIA-derived stool peptidomic signature and machine learning

**DOI:** 10.3389/fmolb.2026.1768474

**Published:** 2026-04-13

**Authors:** Elmira Shajari, David Gagné, Mandy Malick, Patricia Roy, Jean-François Noël, Hugo Gagnon, Maxime Delisle, François-Michel Boisvert, Marie A. Brunet, Jean-François Beaulieu

**Affiliations:** 1 Laboratory of Intestinal Physiopathology, Faculty of Medicine and Health Sciences, Université de Sherbrooke, Sherbrooke, QC, Canada; 2 Centre de Recherche du Centre Hospitalier Universitaire de Sherbrooke, Sherbrooke, QC, Canada; 3 Department of Immunology and Cell Biology, Faculty of Medicine and Health Sciences, Université de Sherbrooke, Sherbrooke, QC, Canada; 4 Department of Medicine, Faculty of Medicine and Health Sciences, Université de Sherbrooke, Sherbrooke, QC, Canada; 5 Allumiqs, Sherbrooke, QC, Canada; 6 Department of Pediatrics, Faculty of Medicine and Health Sciences, Université de Sherbrooke, Sherbrooke, QC, Canada

**Keywords:** biomarker discovery, DIA mass spectrometry (SWATH), fecal calprotectin gray zone, inflammatory bowel disease, machine learning, nested cross-validation, stool proteomics

## Abstract

**Introduction:**

Monitoring disease activity in inflammatory bowel disease (IBD) is essential for guiding therapy and preventing irreversible tissue damage. Colonoscopy, although the gold standard, is invasive and unsuitable for frequent monitoring, while fecal calprotectin lacks accuracy within its diagnostic gray zone (fecal calprotectin 100–250 μg/g). Stool proteomics offers a non-invasive alternative by directly capturing molecular signatures of intestinal inflammation. We conducted a proof-of-concept study to determine whether stool-derived peptides can accurately classify IBD activity (Active vs. Remission) using a fully unbiased and reproducible nested cross-validation machine-learning framework.

**Methods:**

A total of 174 stool samples from IBD patients were collected and profiled using SWATH-DIA mass spectrometry. Feature selection was performed within the training loops only (Boruta, LASSO, RFE) across repeated subsampling, retaining peptides consistently identified in ≥70% of runs. Stable features were used to train four classifiers (GLMNet, SVM-Radial, SVM-Linear, Naïve Bayes) under inner 5-fold tuning. Outer test folds provided fully unseen evaluation, and model performance was additionally assessed exclusively on gray zone samples extracted from the outer test splits to quantify diagnostic resolution in this clinically challenging subgroup.

**Results:**

Nested cross-validation identified a consensus panel of nine stool-derived peptides from five proteins. Across candidate classifiers, performance was broadly similar, with GLMNet consistently achieving the best trade-off between metrics. For GLMNet, outer-fold mean AUC was 0.93 and balanced accuracy 0.88, with specificity 0.94, sensitivity 0.82, and F1-score 0.85; close agreement between inner- and outer-fold metrics indicated minimal overfitting. Within the calprotectin gray zone subgroup (n = 34), GLMNet maintained good performance (balanced accuracy 0.78, F1 0.79, AUC 0.80), confirming that the peptide signature remains informative in this diagnostically challenging range.

**Conclusion:**

A stool-based multi-peptide signature, evaluated with a rigorously nested, leakage-free machine-learning framework, can reliably classify IBD activity and retain discriminative power within the gray zone. This biologically interpretable five-protein panel provides a strong basis for targeted mass-spectrometry assay development and prospective validation as a non-invasive tool for personalized IBD monitoring.

## 1 Introduction

Inflammatory bowel disease (IBD) is a lifelong condition in which the immune system inflames the gut. It mainly appears as two disorders: Crohn’s disease and ulcerative colitis. People with IBD cycle through flares and calmer periods of remission, and treatment often needs to be modified as the disease activity changes. Clinicians need reliable, repeatable tools to monitor whether a patient is inflamed or quiescent, to monitor treatment response over time, and to adjust treatment promptly when needed. Timely recognition of a flare can prevents irreversible mucosal damage and downstream complications, while confirmation of remission can avert overtreatment and its adverse effects (Kucharzik et al., 2023).

Today’s monitoring tools have trade-offs. Colonoscopy allows a direct view on the mucosa, but it is invasive, costly, and not suited for frequent checks. Fecal calprotectin (FC) is a practical stool test used worldwide and is very helpful when values are clearly low or clearly high (Ricciuto and Griffiths, 2019). The problem is the “gray zone” (about 100–250 μg/g) where results are ambiguous; patients are then asked to repeat the test after a few weeks, and many still end up needing endoscopy (West et al., 2023; de Magalhães Costa et al., 2024). Beyond FC, numerous blood- and stool-based biomarkers have been evaluated, yet most lack sufficient discriminatory accuracy or reproducibility for routine clinical use (Kaz and Venu, 2025). This unmet need underscores the value of more informative, non-invasive tests that directly reflect the intestinal inflammatory milieu.

Stool proteomics offers that promise. Proteins and peptides shed into the stool come directly from the inflamed intestinal mucosa, providing a molecular snapshot of what is happening at the mucosal surface. Modern mass spectrometry, especially SWATH-DIA, can measure these signals deeply and reproducibly at scale (Anjo et al., 2017; Sidoli et al., 2015). In our previous work, we showed that stool protein signatures can separate IBD from non-IBD (Shajari et al., 2024) and even distinguish Crohn’s disease from ulcerative colitis (Shajari et al., 2025), suggesting that stool proteomics can support real clinical decisions. Here, we move from diagnosis to monitoring, precisely where current tools struggle most, particularly in the FC gray zone.

The central challenge in analyzing stool samples is their biological heterogeneity, driven by diet, microbiome composition, and inter-individual variability, which makes it difficult to identify stable, generalizable molecular signals (Vujkovic-Cvijin et al., 2020). In this study, we analyzed 174 IBD stool samples, a substantial cohort for proteomics, but a single fixed train–test split would still be vulnerable to sampling variability and yield less robust estimates of feature stability and model performance. To address this, we implemented a machine-learning–based biomarker discovery workflow with nested cross-validation to obtain unbiased estimates of generalization, avoid information leakage, and repeatedly select peptide features that remain consistently informative across resampled training subsets (Vabalas et al., 2019).

Within this framework, our aim was not to deliver a finalized diagnostic classifier, but to derive a stable, non-redundant, and biologically interpretable multi-peptide signature from SWATH-DIA stool peptidomics that can more clearly distinguish Active from Remission IBD patients than current non-invasive tools, with particular attention to the fecal calprotectin gray zone subgroup. By combining transparent feature selection with rigorously nested evaluation, we characterize how this candidate peptide panel behaves both in the overall cohort and within gray zone patients and highlight recurring biological signals that drive model predictions. These results establish the feasibility of this discovery workflow and provide a prioritized set of peptide biomarkers and a methodological foundation for future targeted mass-spectrometry validation and prospective clinical studies.

## 2 Materials and methods

### 2.1 Stool sample collection

Between January 2021 and August 2023, fecal specimens were sourced through the routine fecal calprotectin program of the Clinical Hematology Laboratory at CIUSSS de l’Estrie–CHUS. Participants collected samples at home and delivered them to the hospital within 24 h (up to ∼2 h at room temperature, then refrigerated at 4 °C for ≤24 h). Approximately 50 mg of each specimen was allocated for ELISA-based calprotectin testing; the remaining material was aliquoted and stored at −80 °C for proteomic analyses.

### 2.2 Eligibility criteria and clinical reference

Adults previously diagnosed with Crohn’s disease or ulcerative colitis were eligible when evidence supported either active disease or remission, defined by endoscopic activity in conjunction with consistent clinical history. Where available, histology was considered supportive; however, isolated histological activity without clinical or endoscopic activity was not considered. Samples with uncertain diagnoses were excluded from analysis.

### 2.3 Ethics

All procedures conformed to the Declaration of Helsinki and received approval from the Institutional Research Ethics Committee of CIUSSS de l’Estrie–CHUS (protocol 1991–17, 90–18; latest approval 2025–08-27).

### 2.4 Fecal calprotectin immunoassay

Fecal calprotectin (FC) was quantified by ELISA using Calprest® NG (Ref. 9069) with EasyCal extraction tubes (Ref. 9062), supplied by Qualisys. Sampling followed the kit instructions with the pre-filled extraction solution and standardized stick to ensure reproducible stool loading. Results are reported as µg/g stool over the kit’s 0–3,000 μg/g measuring range according to the manufacturer instructions.

### 2.5 Sample preparation for mass spectrometry

Sample preparation and DIA acquisition followed our previously published protocol (Shajari et al., 2024), with minor adaptations (Shajari et al., 2025). Briefly, ∼100 mg of frozen stool was suspended in lysis buffer (25 mM Tris, 1% SDS, pH 7.5) and centrifuged; protein content in the aqueous phase was quantified by BCA assay. Proteins were reduced with 10 mM DTT, alkylated to 15 mM iodoacetamide, and the reaction was quenched with 10 mM DTT. Precipitation was performed using chilled acetone and methanol. The protein pellet was digested with Trypsin/Lys-C, and resulting peptides were cleaned on Strata-X SPE cartridges, dried at 37 °C for ∼45 min, and stored at −80 °C. Prior to LC–MS/MS, peptides were reconstituted in 20 µL of mobile phase (0.2% formic acid, 3% DMSO in water).

### 2.6 DIA LC–MS/MS acquisition

SWATH-mode LC–MS/MS was carried out as described previously (Shajari et al., 2025). Analyses were performed at the Allumiqs Solutions proteomics facility using an Eksigent μUHPLC coupled to an AB Sciex TripleTOF 6,600 operated in data-independent acquisition (DIA). Peptides were resolved on a reversed-phase column, and isolation window schemes were determined with the Sciex SWATH Variable Window Calculator to optimize coverage.

### 2.7 Spectral library construction and quantitative processing

For library generation, proteins from pooled samples were separated by SDS-PAGE, followed by in-gel digestion and peptide extraction. Data-dependent acquisition (DDA) runs were searched with MSFragger (FragPipe v19.1) (Yu et al., 2023) against the human proteome to build a spectral library comprising ∼2,000 proteins. DIA data were quantified label-free using DIA-NN (v1.8.1) (Demichev et al., 2020) with Match-Between-Run enabled to improve data completeness via accurate mass and retention-time alignment. Protein inference was performed at the gene level with a 1% FDR threshold. Detailed LC–MS/MS parameters, SWATH window settings, solvent compositions, and DIA-NN processing options were as described previously (Shajari et al., 2025).

### 2.8 Statistical analysis for discovering potential biomarkers

**FIGURE 1:**
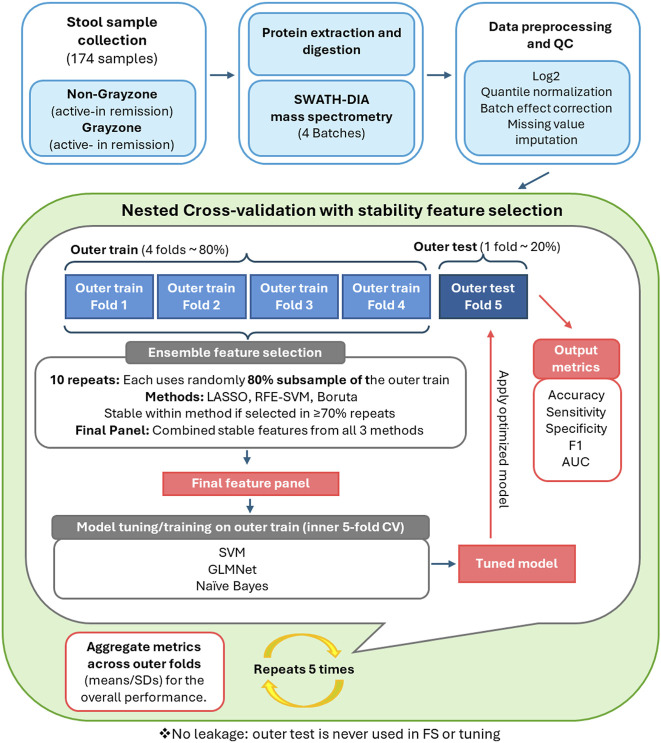
Overview of the study workflow. Stool proteomic profiles were acquired by SWATH-DIA mass spectrometry (four batches) and processed through a standardized preprocessing pipeline. Model development used a leakage-free 5 × 5 nested cross-validation framework, with outer folds stratified to maintain balanced proportions of Active and Remission samples and gray-zone cases. Feature selection was performed within the inner loops using a stability-based ensemble approach (LASSO, RFE-SVM, Boruta), followed by tuning of SVM, GLMNet, and Naïve Bayes models. Performance was evaluated on held-out outer folds and reported as mean ± SD for accuracy, sensitivity, specificity, F1-score, and AUC.

The key steps of the analytical workflow are summarized in Figure 1. All analyses were performed in R 4.2.2 (2022–10-31, UCRT) within RStudio. In total, 174 stool samples were analyzed (66 active IBD, 108 remission), including 34 samples with fecal calprotectin (FC) values in the gray zone (100–250 μg/g). Data preprocessing followed our previously described protocols (Shajari et al., 2024): Raw SWATH-DIA intensities at the protein and peptide levels were log2-transformed and processed as previously described (Shajari et al., 2025). Briefly, samples and features (protein/peptide) with more than 70% missing values were removed, and the remaining data were quantile-normalized (preprocessCore (Bolstad, 2019)). To assess batch structure, we first used exploratory boxplots across the four LC–MS acquisition batches and principal component analysis (PCA), Batch effects were then corrected using ComBat (sva (Leek et al., 2012)), after which intensities were re-inspected by boxplots and PCA to confirm improved alignment across batches. ComBat was applied solely to adjust technical batch effects. Age and sex were not included as covariates in the design matrix, as preliminary analyses indicated no significant association of these variables with disease status or batch assignment.

For the proteome-level description, we first imputed remaining missing values in the batch corrected protein matrix using k-nearest neighbors (impute (Hastie et al., 2011)). For each protein, we then calculated the log2 fold change between Active and Remission samples and performed a two-sample Welch’s t-test. P-values were adjusted for multiple testing using the Benjamini–Hochberg procedure, and proteins with adjusted p < 0.05 and |log2FC| > 0.6 were classified as significantly up- or downregulated; all others were considered non-significant. These statistics were used to generate volcano plots of log2FC versus −log10 (adjusted p-value), with the most significant differentially expressed proteins annotated, and violin plots for selected proteins to illustrate group-wise abundance differences and the degree of overlap between Active and Remission samples.

### 2.9 Feature selection and machine-learning framework using nested cross-validation

All machine-learning analyses were performed at the peptide level, which offers higher analytical resolution, increased feature granularity, and direct compatibility with downstream targeted MRM assay development. To obtain unbiased estimates of model performance and prevent information leakage, we implemented a fully nested cross-validation (CV) framework comprising outer evaluation folds and inner hyperparameter-tuning folds (Cawley and Talbot, 2010).

#### 2.9.1 Outer cross-validation structure

The dataset was partitioned into five outer folds using stratified sampling based on joint disease-activity and calprotectin gray zone status (Active/Remission × Gray/Non-Gray), as shown in Figure 1. This ensured that each fold contained proportional representation of diagnostically challenging gray zone samples. In each iteration, one-fold was held out exclusively for final testing (∼20%; n ≈ 34–35 patients), while the remaining four folds (∼80%; n ≈ 140) constituted the outer-training set.

#### 2.9.2 Feature-selection strategy

To capture complementary signal types and reduce dependence on any single selection algorithm, three methods were applied within each outer-training set: (1) Boruta (Kursa and Rudnicki, 2010) (random forest wrapper with shadow features) to identify all relevant predictors, including potential nonlinear associations; (2) LASSO (Muthukrishnan and Rohini, 2016) (L1-regularized regression) to obtain a sparse and directionally stable coefficient profile; and (3) Recursive Feature Elimination (RFE) (Guyon et al., 2002) with a linear SVM base learner to iteratively remove weak predictors. All selection procedures were executed strictly within the outer-training data. For each outer fold, the selection pipeline was repeated 10 times using 80% subsampling of the outer-training set to assess robustness. Features retained in ≥70% of repetitions were designated as the stable feature panel for that fold and the stable selections from all methods were accumulated to form the final stable feature set for that fold. The outer test fold was not accessed during any step of selection.

#### 2.9.3 Inner cross-validation for hyperparameter tuning

Using only the stable features and the outer-training data, we performed an inner 5-fold CV to optimize hyperparameters for the candidate classifiers, Support Vector Machine (kernlab), GLMNet (glmnet) (Friedman et al., 2010), and Naïve Bayes(klaR) (Weihs et al., 2005) implemented through the caret framework (Kuhn, 2008).

#### 2.9.4 Model training and evaluation

The optimal hyperparameters from the inner loop were used to refit each model on the full outer-training set. The resulting model was then applied once to the corresponding held-out outer test fold to obtain unbiased predictions. Performance metrics included Accuracy, Sensitivity, Specificity, F1-score, and AUROC.

#### 2.9.5 Performance aggregation

The full nested procedure was repeated across all five outer folds, and final model performance is reported as the mean ± standard deviation across outer iterations.

## 3 Results

### 3.1 Patient demographics and stool sample distribution

A total of 174 stool samples were analyzed, including 66 from patients with active disease and 108 in remission. Disease location was heterogeneous, comprising 55% ileal, 21% colonic, and 24% ileocolonic involvement. Overall, 34 samples (19.5%) fell within the fecal calprotectin gray zone (100–250 μg/g) (Active = 21, Remission = 13), and 140 were outside the gray zone (Active = 45, Remission = 95). The age range of participants was from 18 to 90 years, with a mean age of 49.9 years. The sex distribution was nearly equal across both groups, with 58% female and 42% male.

### 3.2 FC result

**FIGURE 2:**
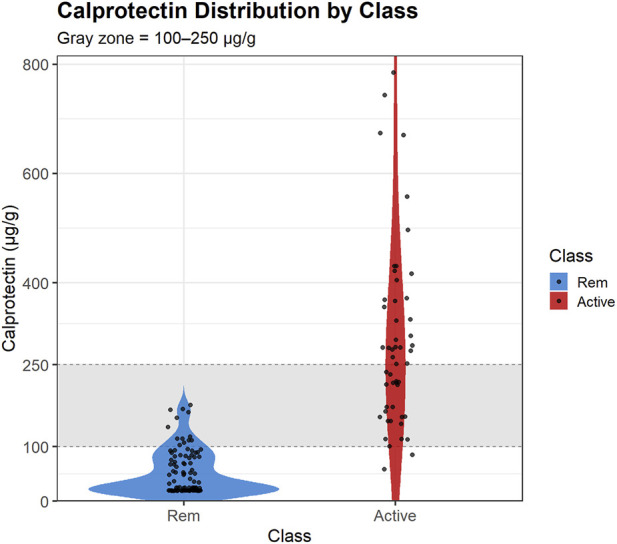
Clinical fecal calprotectin by disease activity class. Violin plots show calprotectin concentrations in Remission and Active IBD groups. The shaded band marks the diagnostic gray zone (100–250 μg/g), highlighting the overlap between groups within this range.

In Figure 2, we present a violin plot showing the clinical FC measurements obtained from the hospital laboratory for the Active and Remission groups. As highlighted in the figure, a subset of samples falls within the gray zone range (100–250 μg/g). In total, 34 samples were in this interval, of which 21 were from Active patients and 13 were from patients in Remission. This distribution illustrates that even FC values do not reliably separate Active from Remission states within this clinically important range. These observations emphasize a key limitation of FC as a standalone monitoring tool and underscore the need for more precise and discriminative stool-based biomarkers.

### 3.3 Exploratory peptidomic profiling and data quality

**FIGURE 3:**
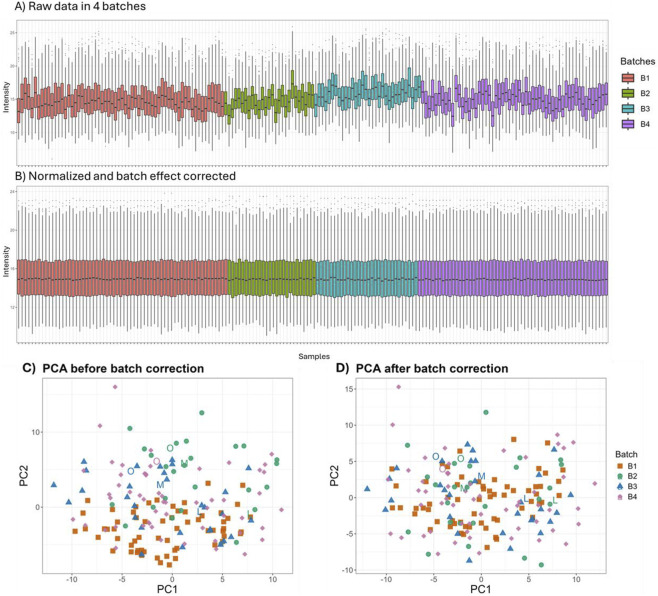
Normalization and batch-effect correction across four SWATH-DIA acquisition batches. **(A)** Boxplots of raw log2 protein intensities show systematic shifts between batches (B1–B4). **(B)** After quantile normalization and batch-effect correction, intensity distributions are aligned across batches. **(C)** PCA before correction shows batch-associated structure, with Batch 1 exhibiting the most apparent separation and labeled replicates more dispersed. **(D)** PCA after correction shows improved overlap among batches and closer clustering of labeled replicates, supporting effective cross-batch harmonization.

Boxplots of log2 intensities across the four acquisition batches revealed clear between-batch differences in the raw data (Figure 3A). After quantile normalization and ComBat batch-effect correction, the intensity distributions across samples became markedly more homogeneous, indicating effective removal of batch-related shifts (Figure 3B). Consistent with these results, PCA of the uncorrected data showed that cross-batch replicate samples, which are expected to lie close together in PCA space, were clearly separated, whereas after ComBat correction these replicates clustered more tightly, indicating that batch-driven offsets were effectively reduced (Figures 3C,D).

**FIGURE 4:**
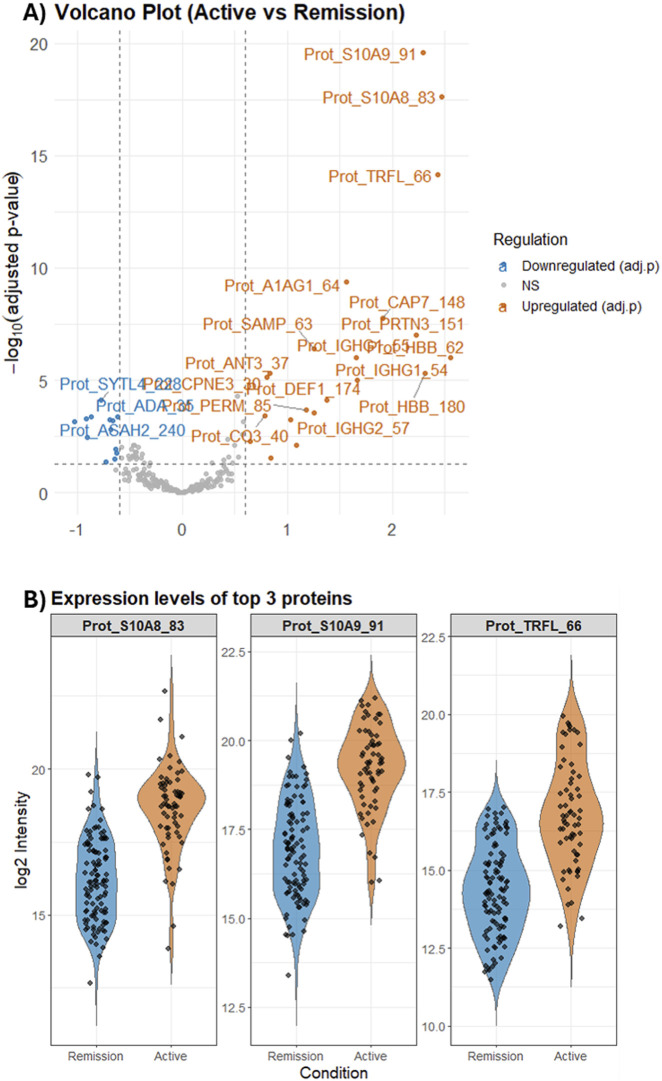
Differential protein abundance between Active and Remission IBD. **(A)** Volcano plot showing log2 fold-change (Active vs. Remission) versus–log10(adjusted p-value); significantly upregulated and downregulated proteins are highlighted. **(B)** Violin plots of log2 intensities for the top three upregulated proteins (S100A8, S100A9, and TRFL) across individual samples, illustrating increased abundance in Active disease but substantial inter-individual overlap between groups.

In this study, we evaluated both protein- and peptide-level datasets. For clarity of presentation, the volcano plots shown here are based on protein-level data, whereas all downstream machine-learning and feature-selection analyses were performed on the peptide-level intensities. As shown in Figure 4A, the protein-level volcano plot identified 20 upregulated and 12 downregulated proteins (based on adjusted *p*-values and fold changes) between Active and Remission IBD. Among these, S10A9, S10A8 and TRFL exhibit the largest fold changes and strongest statistical significance; S10A8/A9 correspond to the two subunits of calprotectin, and TRFL (Lactotransferrin) is a neutrophil-associated protein. In the subsequent Figure 4B, we display violin plots of these three proteins in the two disease-activity groups, which highlight that, despite their significant group differences, there remains substantial overlap in their distributions, indicating that these individual proteins alone are insufficient to cleanly separate Active from Remission samples. This observation further supports the rationale for adopting a multi biomarker strategy, where supervised feature selection and machine-learning models integrate signals from multiple biomarkers to improve the accuracy and robustness of Active versus Remission IBD classification.

### 3.4 Overview of the modeling dataset

We trained peptide-level classifiers to distinguish Active from Remission IBD, including gray zone samples, using the nested cross-validation framework described in method section. The raw file contains 8,400 peptides; after removing those related to contaminants and missing values more than 30% The final dataset contained 670 peptides (related to 175 proteins) in 174 samples after preprocessing.

### 3.5 Feature-selection stability

**FIGURE 5:**
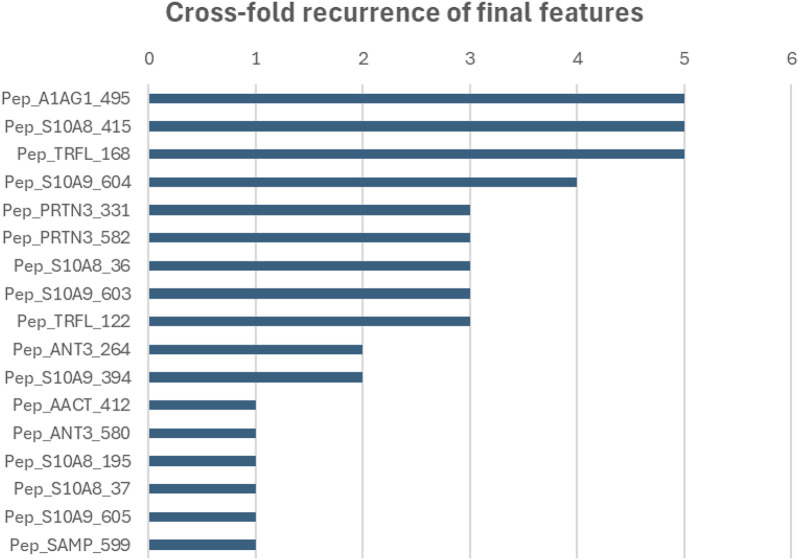
Cross-fold recurrence of the final peptide features. Horizontal bars indicate how many outer cross-validation folds (out of five) selected each peptide in the final stable feature panel derived from inner-loop stability selection. Higher counts reflect peptides that were consistently retained across folds, indicating greater feature-selection stability. Peptides selected in ≥3 folds were retained as the consensus peptide signature panel, reflecting stable feature selection across resampling.

Across the five outer folds, feature selection converged toward a compact multi-peptide panel (Figure 5). The median number of selected peptides per fold was 9 (range: 6–11). A consensus set of nine peptides was selected in ≥60% of all selection repetitions and was carried forward as the stable signature. Eight additional peptides were selected in only one or two folds (mainly from ANT3, AACT, and SAMP) and were not retained. The stable peptides mapped to five predominantly neutrophil-associated proteins (S100A8, S100A9, TRFL, A1AG1, PRTN3) as shown in Table 1.

**TABLE 1:** Consensus peptide panel and annotations. This table lists the peptides selected as the consensus (stable) signature for IBD activity classification.

Peptide_ID	Protein names	Protein description	Genes	Sequence	adj.p	log2FC
Pep_A1AG1_495	A1AG1_HUMAN	Alpha-1-acid glycoprotein 1	ORM1	SDVVYTDWK	0.00	1.82
Pep_S10A8_415	S10A8_HUMAN	Protein S100-A8	S100A8	MLTELEK	0.00	2.54
Pep_S10A8_36	S10A8_HUMAN	Protein S100-A8	S100A8	ALNSIIDVYHK	0.00	2.04
Pep_S10A9_603	S10A9_HUMAN	Protein S100-A9	S100A9	VIEHIMEDLDT(UniMod:21)NADK	0.00	1.88
Pep_S10A9_604	S10A9_HUMAN	Protein S100-A9	S100A9	VIEHIMEDLDTNADK	0.00	2.22
Pep_TRFL_122	TRFL_HUMAN	Lactotransferrin	LTF	EDAIWNLLR	0.00	2.38
Pep_TRFL_168	TRFL_HUMAN	Lactotransferrin	LTF	FFSASC(UniMod:4)VPGADK	0.00	2.36
Pep_PRTN3_331	PRTN3_HUMAN	Myeloblastin	PRTN3	LFPDFFTR	0.00	2.17
Pep_PRTN3_582	PRTN3_HUMAN	Myeloblastin	PRTN3	VALYVDWIR	0.00	2.06

For each peptide, we report the Peptide_ID, associated Protein names, and Genes, together with the Sequence used for identification/quantification. Differential-abundance statistics between Active and Remission groups are provided as adj.p (Benjamini–Hochberg–adjusted p-value) and log2FC (log2 fold change; positive values indicate higher abundance in Active IBD).

### 3.6 Model performance across outer folds

In the nested cross-validation framework, all four classifiers showed consistently strong performance in both the inner tuning loop and the outer held-out test folds (Tables 2 and 3). We employed 5-fold outer cross-validation on the full cohort (n = 174), such that in each outer iteration approximately 80% of samples were used for training and ∼20% (n ≈ 34–35 patients) were reserved as completely unseen test data. Importantly, these outer test samples were not involved in feature selection, hyperparameter tuning, or model training.

**TABLE 2:** Inner cross-validation performance of candidate classifiers.

Glmnet					
Performance metrics	Fold-1	Fold-2	Fold-3	Fold-4	Fold-5
BalancedAccuracy	0.91 ± 0.06	0.88 ± 0.04	0.92 ± 0.04	0.86 ± 0.05	0.89 ± 0.07
Sensitivity	0.87 ± 0.05	0.81 ± 0.06	0.87 ± 0.05	0.79 ± 0.04	0.85 ± 0.14
Specificity	0.95 ± 0.07	0.94 ± 0.04	0.97 ± 0.05	0.93 ± 0.06	0.94 ± 0.04
F1	0.9 ± 0.08	0.85 ± 0.06	0.9 ± 0.05	0.83 ± 0.06	0.87 ± 0.08
AUC	0.94 ± 0.04	0.93 ± 0.04	0.95 ± 0.06	0.93 ± 0.02	0.95 ± 0.06

SVM linear					
	Fold-1	Fold-2	Fold-3	Fold-4	Fold-5
BalancedAccuracy	0.91 ± 0.01	0.86 ± 0.04	0.9 ± 0.04	0.87 ± 0.03	0.88 ± 0.07
Sensitivity	0.89 ± 0.08	0.81 ± 0.11	0.87 ± 0.11	0.81 ± 0.1	0.83 ± 0.13
Specificity	0.93 ± 0.07	0.91 ± 0.07	0.93 ± 0.09	0.93 ± 0.05	0.93 ± 0.06
F1	0.89 ± 0.02	0.83 ± 0.05	0.87 ± 0.06	0.84 ± 0.04	0.85 ± 0.09
AUC	0.95 ± 0.02	0.92 ± 0.06	0.96 ± 0.03	0.93 ± 0.05	0.94 ± 0.06

Naïve bayes					
	Fold-1	Fold-2	Fold-3	Fold-4	Fold-5
BalancedAccuracy	0.89 ± 0.08	0.83 ± 0.05	0.9 ± 0.02	0.84 ± 0.05	0.87 ± 0.05
Sensitivity	0.87 ± 0.11	0.83 ± 0.12	0.87 ± 0.08	0.85 ± 0.08	0.83 ± 0.12
Specificity	0.91 ± 0.06	0.84 ± 0.09	0.93 ± 0.05	0.84 ± 0.08	0.91 ± 0.03
F1	0.86 ± 0.09	0.79 ± 0.06	0.88 ± 0.02	0.8 ± 0.06	0.83 ± 0.05
AUC	0.95 ± 0.03	0.92 ± 0.04	0.96 ± 0.04	0.92 ± 0.07	0.95 ± 0.04

SVM radial					
	Fold-1	Fold-2	Fold-3	Fold-4	Fold-5
BalancedAccuracy	0.9 ± 0.04	0.84 ± 0.08	0.92 ± 0.06	0.87 ± 0.04	0.89 ± 0.09
Sensitivity	0.85 ± 0.11	0.89 ± 0.1	0.87 ± 0.11	0.79 ± 0.08	0.83 ± 0.12
Specificity	0.94 ± 0.04	0.79 ± 0.12	0.98 ± 0.03	0.94 ± 0.06	0.94 ± 0.07
F1	0.87 ± 0.05	0.8 ± 0.08	0.91 ± 0.08	0.84 ± 0.05	0.86 ± 0.11
AUC	0.95 ± 0.04	0.91 ± 0.09	0.94 ± 0.07	0.93 ± 0.04	0.94 ± 0.06

For each outer fold (Fold-1–Fold-5), the table reports the mean ± standard deviation of different metrics obtained in the inner cross-validation loop.

**TABLE 3:** Outer cross-validation performance on held-out test folds.

Glmnet						
Performance metrics	Fold-1	Fold-2	Fold-3	Fold-4	Fold-5	Mean ± SD
Accuracy	0.88	0.97	0.76	0.97	0.89	90 ± 0.09
Sensitivity	0.69	0.92	0.62	1	0.86	82 ± 0.16
Specificity	1	1	0.86	0.96	0.91	94 ± 0.06
F1	0.82	0.96	0.67	0.96	0.86	85 ± 0.12
Balanced accuracy	0.85	0.96	0.74	0.98	0.88	88 ± 0.1
AUC	0.9	1	0.83	1	0.92	93 ± 0.07

Naïve bayes						
	Fold-1	Fold-2	Fold-3	Fold-4	Fold-5	Average
Accuracy	0.82	0.97	0.74	0.92	0.86	86 ± 0.09
Sensitivity	0.69	1	0.62	1	0.86	83 ± 0.17
Specificity	0.9	0.95	0.81	0.87	0.86	88 ± 0.05
F1	0.75	0.96	0.64	0.9	0.83	82 ± 0.13
Balanced accuracy	0.8	0.98	0.71	0.93	0.86	86 ± 0.11
AUC	0.9	1	0.82	1	0.91	92 ± 0.08

SVM linear						
	Fold-1	Fold-2	Fold-3	Fold-4	Fold-5	Average
Accuracy	0.85	0.97	0.74	0.97	0.89	88 ± 0.1
Sensitivity	0.69	0.92	0.62	1	0.86	82 ± 0.16
Specificity	0.95	1	0.81	0.96	0.91	93 ± 0.07
F1	0.78	0.96	0.64	0.96	0.86	84 ± 0.13
Balanced accuracy	0.82	0.96	0.71	0.98	0.88	87 ± 0.11
AUC	0.88	1	0.84	1	0.92	92 ± 0.07

SVM radial						
	Fold-1	Fold-2	Fold-3	Fold-4	Fold-5	Average
Accuracy	0.85	0.94	0.76	0.97	0.86	88 ± 0.08
Sensitivity	0.69	1	0.62	1	0.79	82 ± 0.18
Specificity	0.95	0.91	0.86	0.96	0.91	92 ± 0.04
F1	0.78	0.93	0.67	0.96	0.81	83 ± 0.12
Balanced accuracy	0.82	0.95	0.74	0.98	0.85	87 ± 0.1
AUC	0.88	1	0.86	1	0.9	93 ± 0.07

For each classifier (GLMNet, naïve Bayes, linear SVM, radial SVM), the table reports Accuracy, Sensitivity, Specificity, F1-score, Balanced Accuracy, and AUC, for each outer fold (Fold-1–Fold-5) and the corresponding average across folds.

In the inner cross-validation, mean balanced accuracy typically ranged around 0.86–0.92 with AUC values of approximately 0.92–0.96, indicating robust discrimination between active and remission samples during model selection; GLMNet and radial SVM tended to yield slightly higher and more stable balanced accuracy and AUC than linear SVM and naïve Bayes. In the outer loop, these models generalized well to unseen patients, with mean balanced accuracy of 0.86–0.88 and AUC of 0.92–0.93 across folds. GLMNet achieved the highest average specificity (0.94) and AUC (0.93) while retaining good sensitivity (0.82) and F1-score (0.85), closely followed by the SVM variants, whereas naïve Bayes performed marginally worse, mainly due to lower specificity and F1. The close agreement between inner- and outer-fold metrics supports limited overfitting and motivates the choice of GLMNet as the primary model for downstream interpretation. Across the five outer folds, the strong overlap in misclassified samples, indicating that a substantial fraction of the errors arises from a shared subset of particularly difficult cases rather than model-specific failures, and limiting the potential benefit of ensemble modeling.

### 3.7 ROC and precision–recall analysis of the GLMNet classifier

**FIGURE 6:**
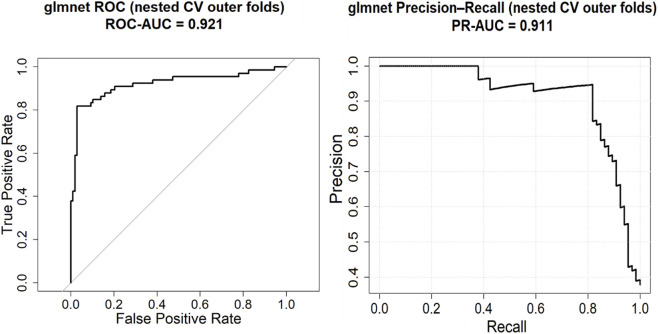
Predictive performance of the GLMNet classifier under nested cross-validation. ROC (left) and precision–recall (right) curves were generated by pooling the predicted probabilities from each outer test fold (i.e., concatenating predictions from all held-out folds) to summarize out-of-sample performance.

To further assess the discriminative behavior of the GLMNet model on unseen samples, we examined pooled ROC and precision–recall (PR) curves derived exclusively from the outer test folds of the nested cross-validation framework (Figure 6). ROC analysis evaluates the model’s overall ranking ability across decision thresholds, whereas PR analysis provides a more informative assessment under class imbalance by emphasizing precision–recall trade-offs. As shown in Figure 6, GLMNet achieved a ROC-AUC of 0.92 and a PR-AUC of 0.91, indicating strong global discrimination and sustained precision across most of the recall range. The precision–recall curve showed near-perfect precision across a broad recall range, with a steeper decline only at very high recall, indicating that false positives accumulated primarily at low probability thresholds.

### 3.8 Gray zone classification performance

Gray zone classification performance was evaluated separately to assess how well the models handled this diagnostically challenging subgroup. Because the number of gray zone samples per outer fold was small, we pooled all gray zone predictions from the outer test folds for each model (34 patients in total) and computed global performance metrics (Accuracy, Sensitivity, Specificity, F1, Balanced Accuracy, and AUC; Table 4). This pooled evaluation provides a more stable and representative estimate of gray zone performance. Overall, the four classifiers produced broadly comparable results, with GLMNet and the linear SVM performing best (Accuracy 0.76, Balanced Accuracy 0.78, F1 0.79, AUC 0.80). The radial SVM showed slightly lower but still robust performance (Accuracy 0.74, Balanced Accuracy 0.76, AUC 0.78), whereas naïve Bayes was mainly limited by reduced specificity (0.69; Balanced Accuracy 0.70; AUC 0.73). Taken together, these results indicate that the learned peptide signature retains meaningful discriminative ability within the gray zone range, particularly for GLMNet- and SVM-based models.

**TABLE 4:** Pooled gray-zone classification performance of the four candidate models.

Model	Accuracy	Sensitivity	Specificity	F1	Balanced accuracy	AUC
Glmnet	0.76	0.71	0.85	0.79	0.78	0.80
Naïve bayes	0.71	0.71	0.69	0.75	0.70	0.73
SVM linear	0.76	0.71	0.85	0.79	0.78	0.80
SVM radial	0.74	0.67	0.85	0.76	0.76	0.78

For the 34 gray-zone patients present in the outer test folds, predictions were pooled across folds and used to compute global Accuracy, Sensitivity, Specificity, F1-score, Balanced Accuracy, and AUC, for each classifier (GLMNet, naïve Bayes, linear SVM, and radial SVM).

**FIGURE 7:**
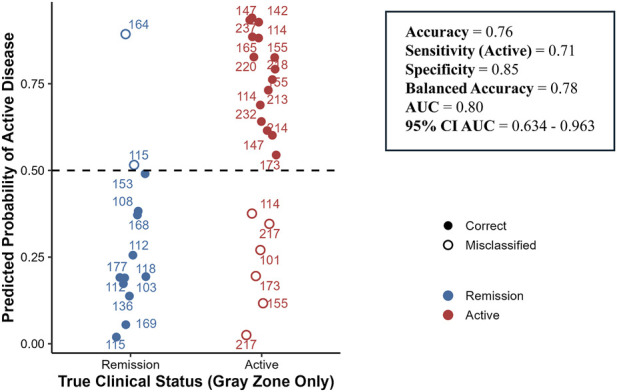
GLMNET model predictions in fecal calprotectin gray-zone samples. Predicted probabilities of active disease generated by the multi-peptide GLMNET model for samples with fecal calprotectin values within the gray zone (100–250 μg/g). The dashed line indicates the 0.5 classification threshold. Filled circles represent correctly classified samples and open circles represent misclassified cases. Numbers shown next to each point indicate the corresponding clinical fecal calprotectin value (µg/g). The model demonstrates enhanced discriminatory performance in this diagnostically challenging subgroup compared to single-marker approaches.

Figure 7 further illustrates GLMNet predictions for gray zone samples. Predicted probabilities are shown relative to the 0.5 decision threshold, with remission cases largely below and active cases largely above this boundary. Although some misclassifications occurred, the distribution demonstrates separation of predicted probabilities despite intermediate fecal calprotectin values (µg/g), which are indicated next to each point.

### 3.9 SHAP-based feature contribution and peptide correlation analysis

**FIGURE 8:**
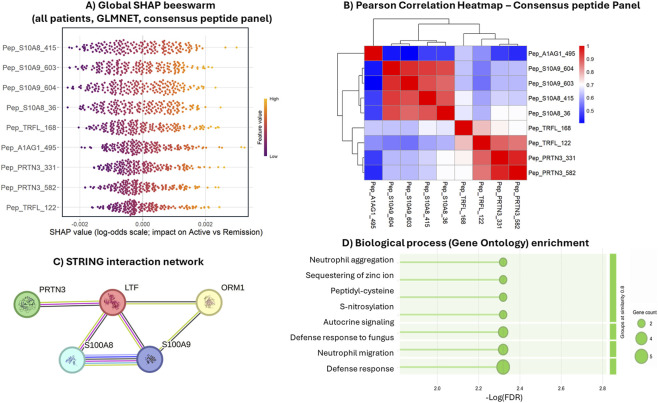
Interpretability and biological coherence of the consensus peptide signature. **(A)** Global SHAP beeswarm plot for the final model, showing the contribution of each consensus peptide to classification (Active vs. Remission) on the log-odds scale; point color indicates relative peptide abundance (low to high). **(B)** Hierarchical clustering heatmap of pairwise correlations among consensus peptides, highlighting strong within-protein co-variation (e.g., S100A8/S100A9 and PRTN3 peptides) and weaker correlations across proteins. **(C)** Protein–protein interaction network of the parent proteins, illustrating known functional associations among the parent proteins based on curated interaction evidence. **(D)** Gene Ontology Biological Process enrichment of the parent proteins, highlighting predominantly immune- and neutrophil-related processes. Dot size indicates the number of contributing proteins, and color reflects enrichment significance, supporting the inflammatory relevance of the consensus signature.

To quantify the contribution of each biomarker within the final signature, we computed global SHAP values using a GLMNet model trained on the consensus peptide panel. GLMNet was selected because it showed slightly better performance than other classifiers in nested cross-validation and provides stable coefficient shrinkage with full compatibility for exact SHAP estimation. The global SHAP beeswarm plot (Figure 8A) demonstrated that higher peptide abundances consistently shifted predictions toward Active disease, whereas lower abundances favored Remission, with stable directionality across patients.

To evaluate relationships among peptides and assess potential redundancy, we calculated pairwise Pearson correlations across the consensus features (Figure 8B). Peptides derived from the same protein showed strong positive correlation, confirming expected within-protein coherence. As anticipated, the two calprotectin subunit peptides (S100A8 and S100A9) also exhibited high correlation. In contrast, correlations between peptides from different proteins were generally low, indicating that each protein contributed distinct quantitative information to the model. We retained two peptides per protein (except for A1AG1) to maintain analytical robustness for downstream targeted mass-spectrometry assay design.

To further characterize functional relationships among the proteins represented in the panel, we generated a protein–protein interaction network using STRING (Figure 8C). The five proteins formed a connected cluster, with the strongest connectivity between S100A8 and S100A9 (calprotectin subunits). LTF and PRTN3 showed multiple interaction edges with the calprotectin proteins, whereas ORM1 (A1AG1) exhibited weaker connectivity, consistent with its role as a more general acute-phase reactant. In Figure 7D, STRING GO Biological Process enrichment highlighted immune-related terms, including broad defense/immune response and more specific neutrophil-related processes. Terms were grouped by similarity to reduce redundancy, suggesting shared immune biology while reflecting partially distinct functions. Overall, these STRING-based results provide functional context indicating that, although the proteins relate to inflammation, they occupy different positions within the network, supporting the non-redundant composition of the final multi-protein signature.

## 4 Discussion

Non-invasive monitoring of inflammatory bowel disease (IBD) currently relies largely on fecal calprotectin (FC) testing. Although FC is widely used and sensitive to intestinal inflammation, its diagnostic performance varies substantially across studies, assay platforms, and selected thresholds, resulting in inconsistent sensitivity and specificity. In general, FC exhibits higher sensitivity than specificity, and this variability has led to the definition of a diagnostic gray zone between 100 and 250 μg/g, where results cannot be reliably interpreted. Patients within this range are often considered diagnostically indeterminate and may require repeat testing or invasive procedures such as colonoscopy, highlighting the need for complementary biomarkers to improve clinical decision-making (Kapel et al., 2023).

In this study, we addressed this gap by identifying a stool-derived multi-peptide signature that complements calprotectin and improves disease activity classification. Using a fully leakage-free nested cross-validation framework, our approach achieved robust performance on held-out test folds, with the selected GLMNet model reaching an overall sensitivity of 0.82 and specificity of 0.94 across all samples (Table 3). Critically, the model also enabled reliable discrimination within the calprotectin gray zone, achieving a sensitivity of 0.71–0.72 and a specificity of 0.85 when predictions were pooled across folds (Table 4).

We identified a stable panel of nine peptides from five proteins including S100A8, S100A9, lactotransferrin (LTF/TRFL), alpha-1-acid glycoprotein 1/Orosomucoid (A1AG1/ORM1), and proteinase 3 (PRTN3). These proteins are largely neutrophil- or acute-phase–associated, highlighting the central role of innate immune activation in mucosal inflammation. Fecal calprotectin and lactoferrin are among the most widely studied inflammatory markers in stool (Dai et al., 2018). Both are released from activated neutrophils, have potent bactericidal and fungicidal activity, and are abundant components of neutrophil granules that are liberated during degranulation and apoptosis. During intestinal inflammation, polymorphonuclear neutrophils migrate into the gut mucosa and lumen, leading to increased fecal calprotectin and lactoferrin concentrations that broadly reflect the extent of neutrophil trafficking into the gastrointestinal tract (Dai et al., 2020). Alpha-1-acid glycoprotein 1 (A1AG1) is a positive acute-phase reactant synthesized predominantly by hepatocytes in response to tissue injury, inflammation, or infection, and its levels are elevated in active compared with inactive IBD (Watanabe et al., 2013; Malicevic et al., 2024). Proteinase 3 (PRTN3), formerly known as myeloblastin, is a neutrophil serine protease stored in azurophilic granules that degrades components of the extracellular matrix and modulates inflammatory signaling at sites of mucosal damage (Kirov et al., 2019). Although several studies have measured these proteins, individually or in combination, they have generally evaluated them as standalone biomarkers or with simple comparative statistics, which has limited their ability to cleanly separate active from quiescent disease, particularly in diagnostically challenging subgroups. In contrast, our machine-learning framework integrates peptide signals from these into a composite multi-peptide signature, enabling more effective discrimination between disease activity states and revealing complementary inflammatory processes that are not captured by any single marker alone.

In the final panel, two peptides belong to an identical amino-acid sequence but differ in charge state and modification status (Table 1). As expected, these features showed a high degree of correlation, reflecting a shared biological origin. Nevertheless, both were retained because DIA quantifies precursor-specific signals, and different charge states or modified forms can exhibit distinct ionization and fragmentation behaviors. Their consistent selection across nested resampling indicates that each precursor contributes complementary and reproducible information for discriminating Active and Remission disease states, rather than representing redundant measurements.

We used ensemble feature-selection through LASSO, RFE-SVM, and Boruta to exploit the strength of each method in peptidomic data. LASSO yields a sparse set of features with clear, directionally consistent linear effects (Tibshirani, 1996), whereas RFE-SVM can identify the peptides that most clearly distinguish between the two clinical groups using margin-based discrimination (Guyon et al., 2002). In addition, Boruta, a robust Random-Forest wrapper, supports these, as it encapsulates non-linear and interaction-driven patterns which are common in biological datasets (Kursa and Rudnicki, 2010). By mixing all three approaches in a consensus framework, we reduce bias of each one and reinforce confidence that the chosen peptides are genuine biological signals, rather than mere methodological artifacts. Because peptide features can be redundant or unstable, we also aimed to retain two representative peptides per protein. More than two adds little new information, whereas a single peptide provides insufficient confidence in protein-level inference. This approach yielded eight peptides mapping to four proteins; α-1-acid glycoprotein was the only exception, represented by a single robust peptide due to common DIA detectability limitations such as instability, missed cleavages, or weak ionization (Bruderer et al., 2015).

Selecting a reliable set of peptides and building a model that truly generalizes is critical for any translational biomarker. For this reason, we used nested cross-validation to obtain realistic performance estimates and to avoid information leakage, a well-known source of overfitting in small or high-dimensional omics studies (Varma and Simon, 2006). By restricting feature selection to the inner loops, each outer test fold effectively represented a genuinely unseen patient cohort. The close agreement between inner and outer performance (AUC ≈0.93) indicates that the model is likely to generalize beyond our dataset. Moreover, repeating feature selection across resampling runs consistently converged on the same nine-peptide panel, supporting that the signature is both stable and biologically reproducible, two essential properties for any biomarker intended for clinical translation.

We applied GLMNet, linear SVM, radial SVM, and Naive Bayes to compare several machine-learning families commonly used in high-dimensional omics analysis. GLMNet was selected because its penalized regression framework produces sparse, interpretable models and handles the strong within-protein peptide correlations effectively. Linear SVM provides a robust discriminative baseline for number of predictors ≫ number of samples data and performs well when linear separation emerges in high-dimensional spaces, while radial SVM was included to examine whether nonlinear boundaries capture additional biological structure. Naive Bayes served as a probabilistic benchmark but as expected, showed lower performance due to its independence assumption, which conflicts with the correlated nature of peptide features.

There are limitations to this study, which need to be considered when interpreting these results. Its 174-patient cohort, although larger than most existing stool proteomic studies, is modest relative to the biological heterogeneity of IBD and the high dimensionality of the peptidomic dataset, and the impact for model stability and generalizability. Our findings therefore constitute proof of concept and not a validated diagnostic instrument. Furthermore, all samples were taken from a single clinical center, suggesting site-specific clinical or technical biases that may not generalize to broader populations. The panel of peptides was itself based on a single DIA dataset which may lead to limited representation of the protein. Finally, while nested cross-validation limits overfitting, the lack of an external validation cohort prevents definitive assessment regarding performance in the real world. Future multi-center studies with patients from larger and more diverse populations, as well as independent validation datasets, will be needed to validate the robustness and translation potential of this proposed peptide signature.

In conclusion, our study illustrates the ability to distinguish Active from Remission IBD, particularly gray zone patients, using stool-derived peptidomic markers, guided by a reliable machine-learning method. We identified a small set of reproducible peptides that capture disease-related signals beyond current non-invasive tests. While these findings serve as a proof of concept, they do support the viability of stool peptidomics as a useful tool for monitoring intestinal inflammation. A subsequent step will be the development of this peptide panel into a targeted mass spectrometry approach such as Multiple Reaction Monitoring (MRM), which provides the specificity and reproducibility critical to clinical application. Larger and multi-center studies will be critical to validate these results and facilitate translation into clinical practice.

## Data Availability

The datasets presented in this study can be found in online repositories. The names of the repository/repositories and accession number(s) can be found below: ProteomeXchange Consortium via the PRIDE partner repository, dataset identifier PXD074117.

## References

[B1] Anjo S. I. Santa C. Manadas B. (2017). Swath‐Ms as a tool for biomarker discovery: from basic research to clinical applications. Proteomics 17 (3-4), 1600278. 10.1002/pmic.201600278 28127880

[B2] Bolstad B. (2019). Preprocesscore A Collect. Pre-Processing Funct. R Package Version 1.46. 0.

[B3] Bruderer R. Bernhardt O. M. Gandhi T. Miladinović S. M. Cheng L.-Y. Messner S. (2015). Extending the limits of quantitative proteome profiling with data-independent acquisition and application to acetaminophen-treated three-dimensional liver microtissues. Mol. and Cell. Proteomics 14 (5), 1400–1410. 10.1074/mcp.M114.044305 25724911 PMC4424408

[B4] Cawley G. C. Talbot N. L. (2010). On over-Fitting in model selection and subsequent selection bias in performance evaluation. J. Mach. Learn. Res. 11, 2079–2107. 10.5555/1756006.1859921

[B5] Dai C. Jiang M. Sun M. J. (2018). Fecal markers in the management of inflammatory bowel disease. Postgrad. Med. 130 (7), 597–606. Epub 20180731. 10.1080/00325481.2018.1503919 30063872

[B6] Dai C. Jiang M. Sun M.-J. Cao Q. (2020). Fecal lactoferrin for assessment of inflammatory bowel disease activity: a systematic review and meta-analysis. J. Clinical Gastroenterology 54 (6), 545–553. 10.1097/MCG.0000000000001212 30994521

[B7] de Magalhães Costa M. H. Sassaki L. Y. Chebli J. M. F. (2024). Fecal calprotectin and endoscopic scores: the cornerstones in clinical practice for evaluating mucosal healing in inflammatory bowel disease. World J. Gastroenterology 30 (24), 3022–3035. 10.3748/wjg.v30.i24.3022 38983953 PMC11230062

[B8] Demichev V. Messner C. B. Vernardis S. I. Lilley K. S. Ralser M. (2020). Dia-Nn: neural networks and interference correction enable deep proteome coverage in high throughput. Nat. Methods 17 (1), 41–44. 10.1038/s41592-019-0638-x 31768060 PMC6949130

[B9] Friedman J. H. Hastie T. Tibshirani R. (2010). Regularization paths for generalized linear models *via* coordinate descent. J. Statistical Software 33, 1–22. 10.18637/jss.v033.i01 20808728 PMC2929880

[B10] Guyon I. Weston J. Barnhill S. Vapnik V. (2002). Gene selection for cancer classification using support vector machines. Mach. Learning 46 (1), 389–422. 10.1023/a:1012487302797

[B11] Hastie T. Tibshirani R. Narasimhan B. Chu G. Narasimhan M. B. biocViews Bioinformatics M. (2011). Package ‘impute’.

[B12] Kapel N. Ouni H. Benahmed N. A. Barbot-Trystram L. (2023). Fecal calprotectin for the diagnosis and management of inflammatory bowel diseases. Clin. Transl. Gastroenterol. 14 (9), e00617. Epub 20230901. 10.14309/ctg.0000000000000617 37440723 PMC10522095

[B13] Kaz A. M. Venu N. (2025). Diagnostic methods and biomarkers in inflammatory bowel disease. Diagnostics 15 (11), 1303. 10.3390/diagnostics15111303 40506875 PMC12154505

[B14] Kirov S. Sasson A. Zhang C. Chasalow S. Dongre A. Steen H. (2019). Degradation of the extracellular matrix is part of the pathology of ulcerative colitis. Mol. Omics 15 (1), 67–76. 10.1039/c8mo00239h 30702115

[B15] Kucharzik T. Verstockt B. Maaser C. (2023). Monitoring of patients with active inflammatory bowel disease. Front. Gastroenterology 2, 1172318. 10.3389/fgstr.2023.1172318 41821818 PMC12952472

[B16] Kuhn M. (2008). Building predictive models in R using the caret package. J. Statistical Software 28, 1–26. 10.18637/jss.v028.i05

[B17] Kursa M. B. Rudnicki W. R. (2010). Feature selection with the boruta package. J. Statistical Software 36, 1–13. 10.18637/jss.v036.i11

[B18] Leek J. T. Johnson W. E. Parker H. S. Jaffe A. E. Storey J. D. (2012). The sva package for removing batch effects and other unwanted variation in high-throughput experiments. Bioinformatics 28 (6), 882–883. 10.1093/bioinformatics/bts034 22257669 PMC3307112

[B19] Malicevic U. Rai V. Skrbic R. Agrawal D. K. (2024). Modulation of Orosomucoid-Like protein 3 activity in the management of inflammatory bowel disease. J. Biotechnology Biomedicine 7 (4), 433–444. 10.26502/jbb.2642-91280167 39619146 PMC11606571

[B20] Muthukrishnan R. Rohini R. (2016). “Lasso: a feature selection technique in predictive modeling for machine learning,” 2016 IEEE international conference on advances in computer applications (ICACA) (Ieee).

[B21] Ricciuto A. Griffiths A. M. (2019). Clinical value of fecal calprotectin. Crit. Reviews Clinical Laboratory Sciences 56 (5), 307–320. 10.1080/10408363.2019.1619159 31088326

[B22] Shajari E. Gagne D. Malick M. Roy P. Noel J. F. Gagnon H. (2024). Application of swath mass spectrometry and machine learning in the diagnosis of inflammatory bowel disease based on the stool proteome. Biomedicines 12 (2), Epub 20240201. 10.3390/biomedicines12020333 38397935 PMC10886680

[B23] Shajari E. Gagne D. Bourassa F. Malick M. Roy P. Noel J. F. (2025). Stool-based proteomic signature for the noninvasive classification of crohn's disease and ulcerative colitis using machine learning. Clin. Transl. Gastroenterol. 16 (11), e00925. Epub 20251101. 10.14309/ctg.0000000000000925 41036804 PMC12637349

[B24] Sidoli S. Lin S. Xiong L. Bhanu N. V. Karch K. R. Johansen E. (2015). Sequential window acquisition of all theoretical mass spectra (Swath) analysis for characterization and quantification of histone post-translational modifications. Mol. and Cell. Proteomics 14 (9), 2420–2428. 10.1074/mcp.O114.046102 25636311 PMC4563725

[B25] Tibshirani R. (1996). Regression shrinkage and selection *via* the lasso. J. R. Stat. Soc. Ser. B Stat. Methodol. 58 (1), 267–288. 10.1111/j.2517-6161.1996.tb02080.x

[B26] Vabalas A. Gowen E. Poliakoff E. Casson A. J. (2019). Machine learning algorithm validation with a limited sample size. PloS One 14 (11), e0224365. 10.1371/journal.pone.0224365 31697686 PMC6837442

[B27] Varma S. Simon R. (2006). Bias in error estimation when using cross-validation for model selection. BMC Bioinformatics 7 (1), 91. 10.1186/1471-2105-7-91 16504092 PMC1397873

[B28] Vujkovic-Cvijin I. Sklar J. Jiang L. Natarajan L. Knight R. Belkaid Y. (2020). Host variables confound gut microbiota studies of human disease. Nature 587 (7834), 448–454. 10.1038/s41586-020-2881-9 33149306 PMC7677204

[B29] Watanabe T. Aoyagi K. Nimura S. Eguchi K. Tomioka Y. Sakisaka S. (2013). New fecal biomarker, Α1-Acid glycoprotein, for evaluation of inflammatory bowel disease: Comparison with calprotectin and lactoferrin. Med. Bull. Fukuoka Univ. 40 (3-4), 155–162.

[B30] Weihs C. Ligges U. Luebke K. Raabe N. (2005). “Klar analyzing German business cycles,” in Data analysis and decision support. Springer, 335–343.

[B31] West J. Tan K. Devi J. Macrae F. Christensen B. Segal J. P. (2023). Benefits and challenges of treat-to-target in inflammatory bowel disease. J. Clin. Med. 12 (19), 6292. 10.3390/jcm12196292 37834936 PMC10573216

[B32] Yu F. Teo G. C. Kong A. T. Fröhlich K. Li G. X. Demichev V. (2023). Analysis of dia proteomics data using msfragger-dia and fragpipe computational platform. Nat. Commun. 14 (1), 4154. 10.1038/s41467-023-39869-5 37438352 PMC10338508

